# P24 Impact of targeted antimicrobial stewardship teaching on clinical pharmacist interventions

**DOI:** 10.1093/jacamr/dlag102.030

**Published:** 2026-06-26

**Authors:** H Bayliss, C Aherne

**Affiliations:** Department of Pharmacy, Addenbrooke's Hospital, Cambridge, UK; Department of Microbiology, Addenbrooke's Hospital, Cambridge, UK; Department of Pharmacy, Addenbrooke's Hospital, Cambridge, UK; Department of Microbiology, Addenbrooke's Hospital, Cambridge, UK

## Abstract

**Background:**

Antimicrobial stewardship (AMS) is a critical component of modern healthcare practice, aiming to optimize antimicrobial use, reduce resistance, and improve patient outcomes (1). Clinical pharmacists play a key role in delivering AMS interventions through reviewing prescriptions, promoting guideline adherence, and supporting safe antimicrobial use. However, variability in confidence, knowledge, and awareness of local initiatives may limit the extent and quality of these interventions. Targeted education offers a potential strategy to strengthen pharmacists’ contributions to AMS (2). This study explores the impact of structured, focused antimicrobial teaching on the frequency and nature of clinical pharmacist AMS interventions within a large teaching hospital.

**Objectives:**

To evaluate the impact of targeted antimicrobial teaching on clinical pharmacist interventions. To improve: (i) the number of AMS interventions pharmacists make; (ii) the quality of AMS interventions that pharmacists make; and (iii) education of trust AMS initiatives within this group of pharmacists.

**Methods:**

The AMS team provided targeted teaching to four clinical pharmacists working in acute medicine over a 10-month period. Hour long teaching sessions occurred monthly with teaching covering common infections and simple AMS initiatives e.g. IV to oral switching. After the teaching period finished, the AMS clinical interventions (I-vents) of this group of pharmacists were reviewed to determine the impact the teaching had on these pharmacists. Data was collected via the Trust’s electronic prescribing system to review their I-vents 6 months prior to the teaching, during the teaching and for 6 months afterwards. Both the number and category were recorded.

**Results:**

Prior to the teaching intervention, pharmacists made an average of two AMS interventions per month. This increased to a peak of eight interventions per month during the teaching period and remained elevated at seven interventions per month for the subsequent six months. A similar trend was observed among all pharmacists. Furthermore, the range of interventions also substantially increased during and following the targeted teaching. Stopping antimicrobial therapy due to a lack clinical indication was a key intervention that occurred after the initiation of these teaching sessions.

**Conclusions:**

Targeted AMS education and training increases the number of I-vents clinical pharmacists make during that training period and remains above baseline for at least six months after. The category of I-vent changes dramatically during and after the teaching with a higher focus on IV to oral switch, penicillin de- labelling, stopping antibiotics and changing antibiotics. The category changes in I-vents are then sustained six months after the training period, showcasing the power of targeted teaching as a strategy to aid antimicrobial stewardships within the clinical pharmacy team.
